# Financial incentives for hypertension control: rationale and study design

**DOI:** 10.1186/s13063-020-4051-7

**Published:** 2020-02-03

**Authors:** Liqiang Zheng, Yali Wang, Sitong Liu, Rui Zheng, Dongmei Pei, Yingxian Sun, Zhaoqing Sun

**Affiliations:** 1 Department of Cardiology, Department of Library and Department of Clinical Epidemiology, Shengjing Hospital of China Medical University, Shenyang, 110004 People’s Republic of China; 2 Department of Respiratory, Shengjing Hospital of China Medical University, Shenyang, 110004 People’s Republic of China; 3 Department of General Medicine, Shengjing Hospital of China Medical University, Shenyang, 110004 People’s Republic of China; 4 Department of Cardiology, Shengjing Hospital of China Medical University, Shenyang, 110004 People’s Republic of China

**Keywords:** Financial incentives, Hypertension, Blood pressure control, Randomized controlled trial, China

## Abstract

**Background:**

Even though the effectiveness of lifestyle modifications and antihypertensive pharmaceutical treatment for the prevention of hypertension and its complications have been demonstrated in randomized controlled trials, the benefits of adhering to these treatments have not been popularized among the general public. Studies suggest that incentive approaches based on behavioral economic concepts can improve patients’ adherence to treatment. Therefore, we aimed to test whether financial incentives will reduce the blood pressure (BP) of hypertensive patients in China.

**Methods/Design:**

This is a multicenter, randomized controlled trial with two parallel arms. A total of 400 participants from six cities in the Liaoning and Shanxi provinces of China are block-randomized into intervention and control group with a 1:1 ratio. Patients in the control group will receive interactive management of mobile devices, including patient education and communication. Patients in the intervention group will receive financial incentives in addition to interactive management of mobile devices, conditional on them achieving their antihypertensive goals or hypertension control. Masking the arm allocation will be precluded by the behavioral nature of the intervention and investigators of BP measurement and statistics are masked to clinic assignment. The primary outcome is net change in systolic BP (SBP) from baseline to month 12 between the intervention and control groups. The secondary outcomes are net change in diastolic BP (DBP), BP control, change in medication adherence and lifestyle, and cost-effectiveness.

**Discussion:**

This trial will determine whether financial incentives will improve hypertension control and generate necessary data for controlling hypertension and concomitant cardiovascular diseases among hypertensive patients in China.

**Trial registration:**

ISRCTN13467677. Registered on 16 May 2019.

## Background

Hypertension is a global public health challenge and a major modifiable risk factor of cardiovascular disease (CVD) and premature death [1]. It has been estimated that 7.6 million premature deaths—54% of stroke and 47% of coronary heart disease worldwide—are attributable to hypertension [2]. In 2013, a study of 17 countries showed that the prevalence, awareness, and control of hypertension were 40.8%, 46.5%, and 32.5%, respectively [3]. The hypertension situation in China is also urgent. Results from the China Hypertension Survey 2012–2015 show that 23.2% (an estimated 244.5 million) of the Chinese adult population (aged ≥ 18 years from 31 provinces in mainland China) had hypertension, 46.9% were aware of their diagnosis, 40.7% were taking prescribed antihypertensive medications, and 15.3% had controlled hypertension [4]. From 2004 to 2015, the total cost of hospitalization for acute myocardial infarction, intracranial hemorrhage, and cerebral infarction reached RMB 153.40, 231.99, and 52.426 billion [5]. Therefore, controlling hypertension has become the top priority in the prevention and treatment of chronic diseases in China.

Several lifestyle-related interventions have been recommended for the prevention and treatment of hypertension. These include weight loss, a diet including lower sodium and higher potassium intake, increasing physical activity, smoking cessation, and reducing excessive alcohol intake [6]. Pharmaceutical antihypertensive treatment can significantly reduce the risk of major CVD events and death [7–10]. Although the effectiveness of antihypertensive treatment and lifestyle modification in the prevention and treatment of hypertension and its complications have been validated in previous studies, this beneficial effect has not been maximized in the general population [6, 11, 12]. The control of hypertension, especially in developing countries, remains insufficient. Medication adherence is an important factor in poor control of hypertension [11]. Therefore, encouraging hypertensive patients to positively change unhealthy lifestyles and to promote regular medication intake is an important challenge that needs to be addressed in current hypertension control. There is an urgent need to identify innovative strategies to improve the treatment compliance of hypertensive patients, further improve the control of hypertension, and reduce the burden caused by hypertension and its complications.

The effectiveness of financial incentives have been validated in many studies. In 2009 [13] and 2016 [14], researchers from the United States and Switzerland found that the rates of smoking cessation in the incentive group were significantly higher than that in the control group. A randomized controlled trial (RCT) concerning financial incentives for weight loss found that participants in both incentive groups lost significantly more weight than participants in the control group. This suggests that incentive approaches based on behavioral economic concepts could have a major impact on reducing the incidence of obesity-related illnesses [15]. Sen et al. used a RCT to test the effectiveness of financial incentives for improving adherence to remote-monitoring regimens among patients with poorly controlled diabetes and concluded that participants in the incentive group had higher monitoring rates, relative to the control group [16]. In 2013, a cluster RCT of hypertension care in the United States also showed that individual financial incentives resulted in greater blood pressure (BP) control or appropriate response to uncontrolled BP [17].

Financial incentives incorporate the principle of behavioral economics. Behavioral economics combines psychology and economics to study how human psychology influence individuals’ decisions concerning economic activities. Loss aversion refers to the tendency to be more motivated to avoid losses than achieve similarly sized gains [18]. The regret theory suggests that people’s choice will be affected by expectations of regret or disappointment; this anticipated regret has been shown to affect a variety of preventive behaviors [15].

Based on the above, we aim to determine whether financial incentives will reduce the BP of hypertensive patients in China. All interventions, including interactive management of mobile device and financial incentives, have been validated in previous studies on hypertension control or other disease indicators. This study will generate the necessary data for controlling hypertension via financial incentives and promoting it on a larger scale will be considered if meets the cost-effectiveness principle.

### Objectives

The overall aim of this study is to explore whether financial incentives will reduce the BP of hypertensive patients’ in China. The specific aims of this RCT are:

1. To test whether financial incentives will lower systolic BP (SBP) and diastolic BP (DBP) among hypertensive patients over a 12-month period, compared to the control group;
2. To evaluate whether financial incentives will increase the control rate of hypertension among hypertensive patients over a 12-month period, compared to the control group; and
3. To estimate the cost-effectiveness of this intervention program compared to control group.

## Methods

### Research design

**Fig. 1:**
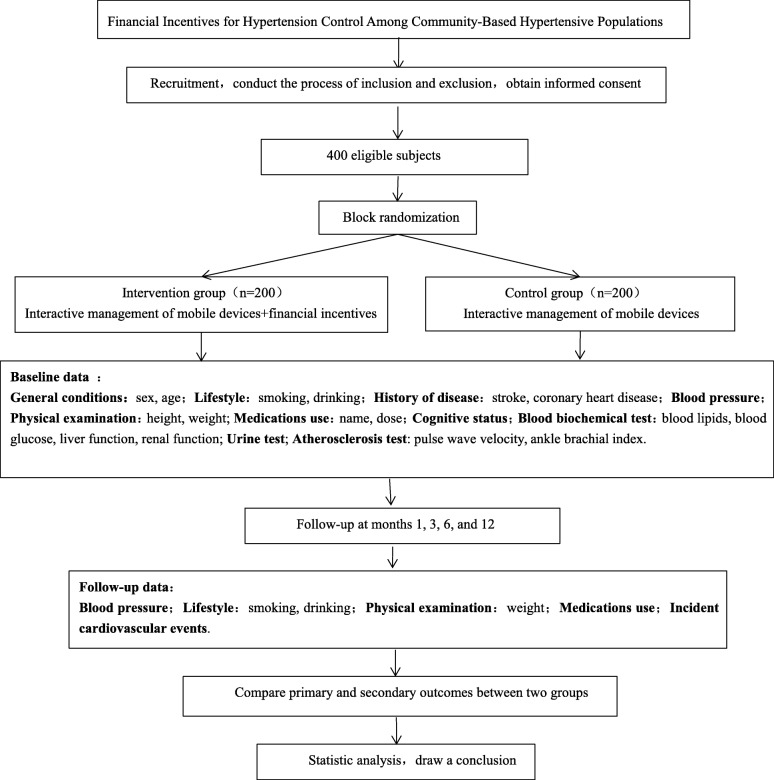
Study design of the project

This study is a multicenter RCT. A total of 400 hypertensive patients from six cities in the Liaoning and Shanxi provinces of China will be recruited. Of these, 200 participants will be assigned to the intervention group and 200 participants to the control group by block randomization. The financial incentives will last for 12 months (Fig. 1). The primary outcome is the net change in SBP from baseline to month 12 between the intervention and control groups. We will also conduct health economic analysis and consider promoting it on a larger scale if meets the cost-effectiveness principle.

### Study population

Patients of this trial will be selected from six cities in Liaoning and Shanxi provinces in China. The institutions for selection include Shengjing Hospital of China Medical University, Fushun Mining General Hospital of Liaoning Health Industry Group, Dandong Central Hospital, Anshan Central Hospital, Fuxin Mining General Hospital of Liaoning Health Industry Group, and Fenyang Hospital of Shanxi Medical University. The inclusion and exclusion criteria for the study population are listed below.

The inclusion criteria are as follows:

1. Aged 35–75 years;
2. Hypertensive patients (SBP/DBP ≥ 140/90 mmHg during two separate screening/baseline visits);
3. Have WeChat and use it skillfully;
4. Local residents who have been living in the city for > 12 months;
5. Voluntarily joined and signed informed consents.

The exclusion criteria are:

1. Pregnant women or women who are planning to become pregnant during the next year;
2. Patients with plans to move from their place of residence during the next year;
3. Individuals with malignant tumors or severe liver or kidney dysfunction;
4. Secondary hypertension patients.

All study participants meeting the inclusion and exclusion criteria will be additionally assessed by a trusted member of the staff.

The research protocol was approved by Shengjing hospital of China Medical University Medical Ethics Committee (No. 2019PS397K) and written informed consent was signed by all the participants or their guardians during screening.

### Randomization and blinding

Patients will be block-randomized to the intervention and control groups in a 1:1 ratio by an independent statistician using a computer-generated randomization list. The randomization will take place after the patients have been enrolled. The randomization is stratified by geographic regions and the number of stratification is 6. Due to the behavioral nature of the intervention, patients and principle investigators will not be blinded to patient allocation. However, investigators responsible for BP measurement and statistics will be masked to clinic assignment. The details of the randomization will be kept confidential until completion of data analysis.

### Intervention

We will explore whether financial incentives will reduce the BP in hypertensive patients in China. Participants in the control group will receive interactive management of mobile devices, including the following:

1. Patient education: researchers will send relevant knowledge concerning prevention and treatment of hypertension at the beginning of each week using WeChat, including the definition and severity of hypertension, methods for correctly measuring BP, lifestyle-related hypertension prevention, the types and selection of antihypertensive drugs, and the harmful effects and complications of hypertension.
2. Communication: if the patients have any questions during the intervention and follow-up, they can communicate with the researcher through the mobile at any time and the researcher will answer their questions.

Patients in the intervention group will receive financial incentives in addition to interactive management of mobile devices. The specific measures include two parts:

1. Participants will be encouraged to self-measure BP once a week and record the condition of antihypertensive medications. A reward of RMB 5 (red envelope) per participant will be awarded if they record the results accurately and completely every week.
2. At the follow-up months 1, 3, 6, and 12, researchers will measure participants’ BP and give a reward of RMB 50 or an equivalent gift (shopping cards, phone bills, cooking oil, etc.) to the patients whose BP values were decreased by 10 mmHg (compared to their baseline BP values) or achieve the control criteria (SBP/DBP < 140/90 mmHg).

### Study outcomes

The primary outcome is net change in SBP, defined as the difference between the intervention and control groups from baseline to follow-up months 12. Secondary outcomes include net change in DBP, the proportion of hypertension control (BP < 140/90 mmHg), changes in self-reported medication adherence, body mass index (BMI), physical activity, counselling frequency, counselling content, number of steps in WeChat, pulse wave velocity (PWV), ankle brachial index (ABI), and cost-effectiveness among all participants.

### Data collection

Data on demographics (sex, age, ethnicity, education level), lifestyle (smoking, alcohol drinking, salt, physical activity), medical history, use of antihypertensive medication and concomitant drugs (hypoglycemic drugs, lipid-lowering drugs, antiplatelet drugs and anticoagulant drugs, etc.; name, dose, adherence, cost), cognitive status, atherosclerosis examination (PWV, ABI), and laboratory tests will be obtained by trained researchers through standardized questionnaires and measurement (Table 1). Two visits—at baseline and at 12-month termination—will be conducted to obtain repeated BP measurements. Current smoking is defined as smoking at least one cigarette per day and lasting for at least one year. Current drinking is defined as ≥ 2 drinks/week and lasting for at least six months. Physical activity is defined as > 30 min/day of moderate physical activity. The patients will be asked whether they exercise > 30 min per day. If the answer is yes, they will be asked what kind of physical activity they do (running, swimming, fitness, fast walking, cycling, etc.) and the average days per week. Salt and cooking oil are defined as the average salt in home in the past year (g/month).

**Table 1:** Data collection schedule

Items	Baseline 1	Baseline 2	Follow-up time (months)				
			1	3	6	12(1)	12(2)
Informed consent	×						
Inclusion and exclusion process	×	×					
Physical examination	×		×	×	×	×	
Lifestyle	×		×	×	×	×	
History of disease	×						
Cognitive status	×						×
Blood pressure	×	×	×	×	×	×	×
Medications use	×		×	×	×	×	
Blood and urine examination		×					×
Atherosclerosis tests		×					×
Incident CVD events			×	×	×	×	

Baseline 1 and Baseline 2 represent the first and second day of the baseline survey, respectively

12 (1) and 12 (2) represents the first and second day of follow-up months 12

*CVD* cardiovascular disease

Each of the BP measurements will be obtained by study nurses who are masked to clinic assignment and measurement will be according to the standard protocol recommended by the American Heart Association [19]. BP will be measured with the participant in a seated position after 5 min of quiet rest. Additionally, participants will be advised to avoid alcohol, cigarettes, coffee/tea, and exercise for at least 30 min before their BP measurement. At the same time, the condition of antihypertensive medications will also be asked before measurement. BP is measured three times on one day with a 1-min interval and the mean of six BP values was calculated and used for all subsequent analysis. BP was measured using a standardized automatic electronic sphygmomanometer (HEM-8102A; Omron, Tokyo, Japan) and one of four cuff sizes (pediatric, regular adult, large, or thigh) will be chosen on the basis of each participant’s arm circumference.

Anthropometric measurements were measured with participants wearing light clothing and without shoes by investigators using a standard protocol. Three measurements will be obtained at each visit for weight, height, waist circumference, and hip circumference; the arithmetic means will be used for analyses.

### Data management and quality control

All individuals in the study cohort will be uniquely encoded according to uniform rules. The data from the epidemiological questionnaire and clinic examination will be reviewed by the quality control specialist. All the investigators will receive training before the trial begins and all the measurements will be obtained using standard methods. All records will be stored securely and confidentially according to standard guidelines. A reason must be indicated when data are altered and all alterations will be saved. To improve response rates, all participants will receive a participation bonus of RMB 100 per person at baseline and a transportation subsidy of RMB 50 per follow-up.

### Safety monitoring and assessment

The researchers will ask patients to report adverse events and the patient’s BP will be continuously monitored. If participants are monitored for excessive BP reduction, the study coordinator will contact the participant to inquire about their health status. This part will be evaluated by specialists in cardiology.

### Data analysis and statistical power

Baseline characteristics (demographics, lifestyle factors, BP, laboratory measurements, and history of disease) will be compared between the intervention and control groups using the independent sample t-test or χ^2^ tests. The mixed-effects model will be used to compare the primary outcome that there is a greater reduction in BP in the intervention group than in the control group. In this model, participants and clinics will be assumed to be random effects and intervention group, time, and the interaction will be assumed to be estimable fixed effects. Logistic regression will be used to analyze the categorical variables. As the measurement of the participants will be carried out at multiple time points, the generalized estimating equation (GEE) will be used for analysis. The proportion of each variable in promoting the generation of outcome will be used for the mediation analysis.

The proposed trial is designed to provide 90% statistical power to detect a ≥ 7.0 mmHg reduction in SBP at the end of the trial, a significance level of 0.05 using a two-sided test and standard deviation (SD) of 20.0 mmHg [20].

## Discussion

The primary aim of this trial is to test the effectiveness of financial incentives on lowering BP and hypertension control among hypertensive patients. An important theoretical basis in incentive studies is behavioral economics. The behavioral economics theory suggests that an important factor of non-adherence is that patients do not perceive a clear cause-and-effect relationship between non-adherence and the increased likelihood of disease progression. Therefore, many patients do not internalize the consequences of non-adherence until it is too late [21, 22]. Financial incentives are behavioral economic interventions that involve applying incentives to participants rather than providers. This method has great potential to change participant health behaviors [15].

The intervention of this trial is providing financial incentives to patients who achieve the goals of lower BP or hypertension control. Financial incentives target two major aspects: medication adherence and self-measuring of BP. The benefits of any commonly used antihypertensive medications on reducing the risk of CVD and deaths have been demonstrated; however, these benefits have not been popularized in the general population [6–12]. The barriers to BP control are primarily related to therapy adherence. Adherence to antihypertensive medications is difficult because they are costly, prone to side effects, and no benefits are immediately observed [23]. Medication adherence is an important factor in poor control of hypertension [11]. Therefore, we believe that financial incentives as a means of promoting patient adherence will significantly improve the control of hypertension.

Self-measurement of BP in patients is part of a self-management strategy that is effective in combination with other co-interventions [24]. Studies have shown that self-monitoring of BP can lead to BP control that is at least as good as office-monitored BP; it can also result in slightly better control, possibly because of better adherence to treatment [25]. In this trial, we encourage patients to measure BP at least once a week with the aim of increase their awareness of their BP and thereby their compliance with treatment.

Patients in the control group of this trial will receive interactive management of mobile device, which targets the patient education and communication. A crucial factor in poor hypertension control is inadequate cognition of the risk of hypertension among patients [12]. Mobile technology offers a new approach to the study of cardiovascular health and has been explored in previous studies [26–28]. Wechat, a popular communication platform in China, has become an indispensable part of life for most adults. During the trial, we will send relevant information concerning prevention and treatment of hypertension using Wechat, which is a more attractive form of communication for most modern Chinese individuals. Patients will be correctly informed about what hypertension is, what the risks of hypertension are, and what the benefits of antihypertensive therapies are. This information is vital for the prevention and treatment of hypertension.

The above discussion illustrates the rationale and consideration of this trial. In summary, this trial will provide a rigorous assessment of this intervention and will be performed under a strict quality control and supervision system. This is the first study to use financial incentives to increase the control rate of hypertension in China to date, which will generate necessary data aimed at control of hypertension, and consider promoting it on a larger scale if it meets the cost-effectiveness principle (Additional file 1).

## Trial status

Protocol version 1.0 and dated 11 November 2018. Recruitment to this study began in May 2019, which is expected to end in July 2020.

## Supplementary information

**Additional file 1.** SPIRIT 2013 Checklist: Recommended items to address in a clinical trial protocol and related documents.

## Data Availability

The datasets used and/or analyzed during the current study are available from the corresponding author on reasonable request.

## Abbreviations

ABI: Ankle brachial index
BMI: Body mass index
BP: Blood pressure
CVD: Cardiovascular disease
DBP: Diastolic blood pressure
GEE: Generalized estimating equation
PWV: Pulse wave velocity
SBP: Systolic blood pressure
SD: Standard deviation
